# Acute liver failure due to herpes simplex virus: diagnostic clues and potential role of plasmapheresis: A case report

**DOI:** 10.1097/MD.0000000000027139

**Published:** 2021-09-03

**Authors:** Sebastián M. Chávez, Jaime M. Poniachik, Álvaro M. Urzua, Juan P. Roblero, Máximo J. Cattaneo, Andrea P. Jimenez, Laura E. Carreño, Rodrigo A. Cornejo

**Affiliations:** a Unidad de Pacientes Críticos, Departamento de Medicina, Hospital Clínico Universidad de Chile. Santiago, Chile; b Sección Gastroenterología, Departamento de Medicina, Hospital Clínico Universidad de Chile. Santiago, Chile; c Departamento de Anatomía patológica, Hospital Clínico Universidad de Chile. Santiago, Chile.

**Keywords:** acute liver failure, case report, hepatitis, herpes simplex virus, plasmapheresis

## Abstract

**Introduction:**

Acute liver failure (ALF) is a life-threatening condition that remains challenging for physicians despite several advances in supportive care. Etiologies vary worldwide, with herpes simplex virus (HSV) hepatitis representing less than 1% of cases. Despite its low incidence, ALF is a lethal cause of acute necrotizing hepatitis and has a high mortality. Early antiviral treatment is beneficial for survival and decreased liver transplantation necessity. However, plasmapheresis, despite its theoretical potential benefit, is scarcely reported.

**Patient concerns:**

A 25-year-old woman with no known disease presented with painful pharynx ulcers, increased transaminases and impaired liver function.

**Diagnosis:**

ALF due to a disseminated HSV-2 primary infection was diagnosed with a positive polymerase chain reaction for HSV-2 in the biopsied liver tissue and blood.

**Interventions:**

Empiric antiviral treatment was initiated. After clinical deterioration, plasmapheresis was also initiated.

**Outcomes:**

After 6 cycles of plasmapheresis and supportive care, the patient's condition improved without undergoing liver transplantation.

**Conclusions:**

ALF is a life-threatening condition, and HSV as an etiology must be suspected based on background, clinical manifestation, and laboratory information. The potential role of plasmapheresis in HSV hepatitis should be considered.

## 1 Introduction

Acute liver failure (ALF) is a rare but life-threatening condition characterized by acute liver injury, hepatic encephalopathy, and coagulopathy.^[1]^ The original term, fulminant liver failure, was defined in the 70 second as a severe liver injury that is potentially reversible in nature and induces hepatic encephalopathy within 8 weeks of the onset of first symptoms in the absence of pre-existing liver disease.^[2]^ Subsequently, multiple classification systems and phenotypes have been described,^[3]^ including those with previous liver disease. Despite progress in classification systems, mortality remains high.^[4]^ Therefore, early recognition and multidisciplinary management of patients are crucial.

A wide variety of etiologies can cause acute liver failure;^[5]^ however, the exact incidence is unknown and there are regional differences. Overall, viral and drug-induced hepatitis are considered the most common ALF causes.^[6]^ Among viral etiologies, both herpes simplex virus (HSV)-1 and HSV-2 have been documented. Despite a high seroprevalence in the general population,^[7]^ ALF is a rare complication of HSV. ALF has been described mostly in neonates, immunosuppressed patients,^[8]^ and pregnant women,^[9]^ with poor outcomes.^[10]^

We present the case of an immunocompetent patient with ALF due to a disseminated HSV-2 primary infection who was successfully treated with antiviral medications, plasmapheresis, and supportive care. We review the updated literature and discuss clinical clues for early recognition, diagnosis, and treatment options besides hepatic transplantation.

## 2 Case report

**Figure 1:**
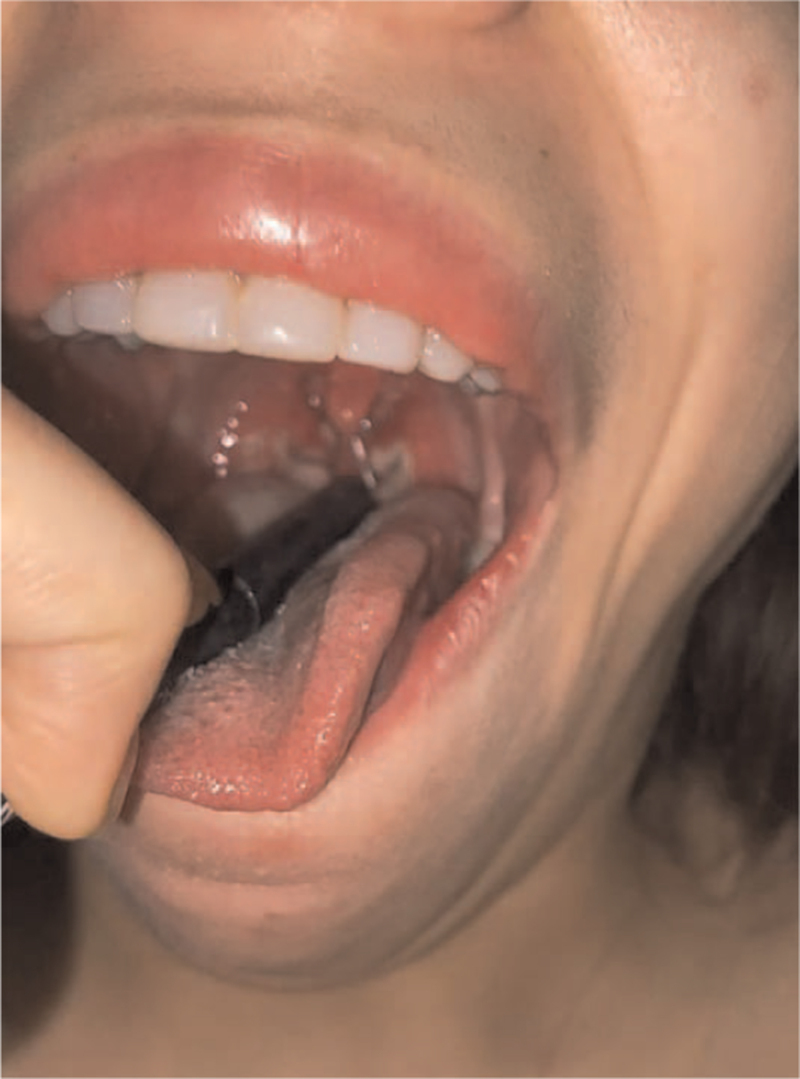
Pharyngitis with ulcers on both sides.

A 25-year-old woman presented to the emergency department with a 9-day history of malaise, fever up to 40°C, chills, and odynophagia. She reported losing approximately 2 kg weight during the last 2 weeks. At admission, she was dehydrated, afebrile, and normotensive but was also tachycardic. Physical examination revealed a congested and erythematous pharynx associated with painful ulcers and cervical tender lymphadenopathy (Fig. 1). Abdominal examination revealed painful hepatosplenomegaly. No jaundice or other skin lesions were found.

She was originally from Venezuela and lived in Chile for 4 years. She had no known diseases, and her medical history was unremarkable; however, gynecological surgery was performed 3 weeks earlier for a hemorrhagic corpus luteum cyst. Biopsy was confirmatory, and she was discharged satisfactorily after a good postoperative period. She had no known allergies and denied the consumption of alcohol, supplements, vitamins, tobacco, or any illicit drugs. She was never pregnant, denied previous sexually transmitted infections, and had 1 sexual partner for years, without using any barrier or contraceptive method. There was no relevant familial history of any disease. She did not consume any medication apart from the medication prescribed recently after the aforementioned surgery, which was 1 g of acetaminophen every 6 hours for 1 week. When she started with odynophagia, she visited an ambulatory clinic and was diagnosed with bacterial pharyngitis; benzathine penicillin was administered, followed by amoxicillin with clavulanic acid, until she visited our hospital again as her symptoms persisted.

Laboratory tests were performed (Table 1). Values were significant for mild anemia (Hb: 10.6 g/dL). She had no leukocytosis and had lymphopenia with a lymphocyte count of 774 cells/uL and a platelet count of 607 k/uL. She had elevated C-reactive protein levels (84 mg/dL), and kidney function was preserved. Her alanine aminotransferase (ALT) and aspartate aminotransferase (AST) levels were elevated (354 U/L and 361 U/L, respectively), and her alkaline phosphatase (AP) and total bilirubin levels were normal. Her international normalized ratio was mildly elevated (1.29). She was diagnosed with mononucleosis syndrome and was hospitalized for further observation.

**Table 1:** Laboratory results on admission and in-hospital progression.

	Normal ranges	3 weeks earlier (Surgery)	On admission	72 hours	Day N° 5 (1° TPE)	Day N° 11 (6° TPE)	Day N° 78 (Discharge)
Hb	12–16 g/dL	11.1	10.6	9.3	9.6	8.2	9.2
WBC	4–10 × 1000/uL	16070	9680	2070	7600	14170	16100
Plat	150–400 × 1000/uL	281000	607000	182000	28000	67000	744000
TB	0.2–1.3 mg/dL	0.65	0.43	1.67	4.73	7.26	0.59
AP	38–126 U/L	43	100	331	285	92	769
GGT	12–43 U/L		140	334	231	72	992
AST	14–36 U/L	16	361	6776	5684	158	73
ALT	0–35 U/L		354	3870	3058	128	80
LDH	120–340 U/L	194		9590	2492	647	401
INR		1.12	1.29	1.53	2.49	1.68	1.25
Fib	180–350 mg/dL			287	95	142	179^∗^
F V	50%–150%			16%		36%	41%^†^
F VII	50%–150%			17%			
F VIII	50%–150%			67%			

ALT = alanine aminotransferase, AP = alkaline phosphatase, AST = aspartate aminotransferase, F V = factor V, F VII = Factor VII, F VII = factor VIII, Fib = fibrinogen, GGT = gamma-glutamyl transpeptidase, Hb = hemoglobin, INR = international normalized ratio, LDH = lactate dehydrogenase, Plat = Platelets, TB = total bilirubin, TPE = Total plasma exchange, WBC = white blood cells.

∗ Last sample on day 46.

† Last sample on day 31.

**Figure 2:**
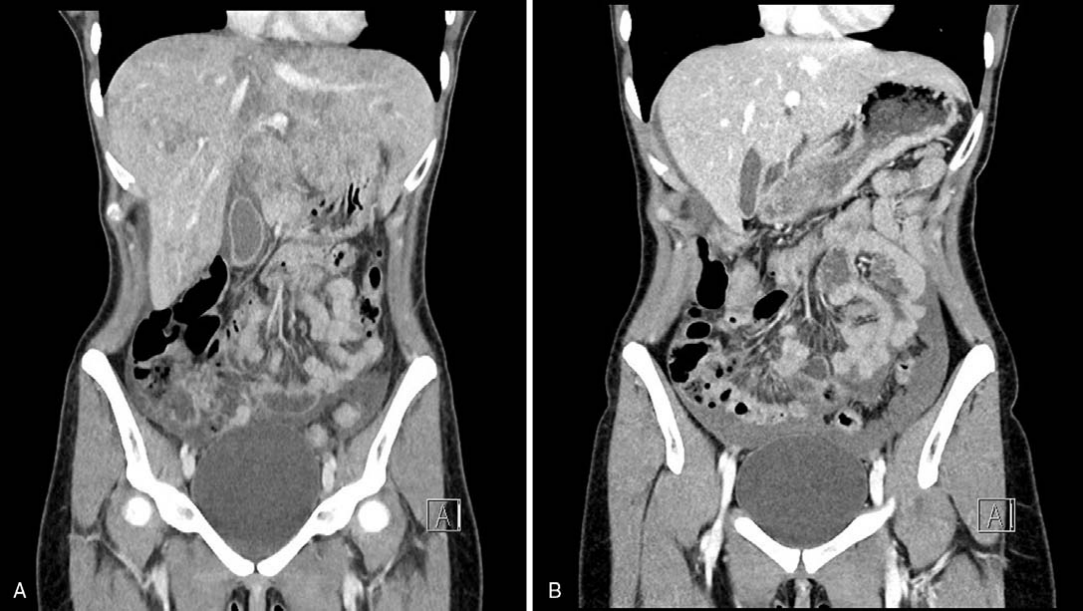
(A) Abdominal computed tomography (CT) when abdominal pain developed. Hepatosplenomegaly and edema of the gallbladder wall and liver parenchyma, hepatic hilar adenopathy, and ascites, findings suggestive of acute hepatitis were apparent. (B): Abdominal CT performed 3 weeks earlier during gynecological surgery. The liver and spleen are normal.

After admission, the patient's clinical status declined. On the third day, she had a fever and severe abdominal pain, and an abdominal computed tomography was performed (Fig. 2). The laboratory tests (Table 1) showed a marked increase in transaminases and new coagulopathy. She was transferred to the intensive care unit (ICU). Upon admission to the ICU, there were no signs of encephalopathy or jaundice. Based on clinical suspicion, acyclovir and N-acetylcysteine were empirically initiated alongside supportive care.

The patient's hepatic insufficiency progressed. On the 4th day, she developed clinical jaundice, and on the 6th day, encephalopathy was evident, and she required intubation. Coagulopathy progressed; the patient had 16% V factor, 17% VII factor, 90 mg/dL fibrinogen levels, 2.5 international normalized ratio, and 57 seg activated partial thromboplastin time, requiring daily blood-derived transfusion. Broad etiologic studies were performed (Table 2), including a transjugular hepatic biopsy. A blood polymerase chain reaction (PCR) for HSV-2 and quantitative IgM was positive. Hepatic biopsy showed necrotizing hepatitis, and immunohistochemistry and tissue PCR were positive for HSV-2 and negative for CMV. A HSV-2 PCR test for pharynx ulcers and persistent pleural effusion was also positive. Fulminant liver failure due to a disseminated HSV-2 primary infection was diagnosed. IV acyclovir was administered, and plasmapheresis was initiated when hepatic encephalopathy started.

**Table 2:** Etiologic studies.

Etiologic studies	Results
Antistreptolysins	Normal
Paul Bunnel reaction	Negative
Epstein Barr Virus IgM/ Epstein Barr Virus IgG Blood Epstein Barr Virus PCR	Negative Positive Negative
Blood CMV PCR CMV IgM CMV IgG	Negative Negative Positive
HBsAg, AntiHBc	Negative
Anti HAV IgM Total anti HAV	Negative Positive
Anti HCV IgG	Negative
First: HSV-2 IgM/IgG Second: Pharynx HSV-2 PCR Liver tissue HSV-2 PCR Blood HSV-2 PCR Pleural effusion HSV-2 PCR	Inconclusive (0.98)/Inconclusive (1.0) Positive Positive Positive Positive
HSV-1 IgM/IgG	Negative/Negative
VZV IgG	Positive
IgA/IgM/IgG (mg/dL)	Normal (277/1320/272 mg/dL, respectively)
ANA, AMA-M2, M2–3E, Sp100, PML, gp210, LKM1, LC-1, SLA/LP, Ro-52	Negative
Bone marrow flow cytometry Mielogram	Negative for lymphoproliferative disorders Erythroid hypoplasia
Ferritin Seric Fe Saturation coefficient	>40000 ng/mL 16 ug/dL 17%
HIV	Non-reactive
VDRL	Non-reactive
B-hCG	Negative
Blood PCR Parvovirus B19	Negative

AMA-M2 = antimitochondrial antibodies, M2 subtype, ANA = antinuclear antibody, B-hCG = human chorionic gonadotropin beta-subunit, CMV = cytomegalovirus, HAV = hepatitis A virus, HCV = hepatitis C virus, HIV = human immunodeficiency viruses, HSV = herpes simplex virus, LC-1 = anti-liver cytosol antibody, LKM1 = anti-liver-kidney microsomal-1, M2-E3 = AntiM2-E3 Elisa IgG, PCR = polymerase chain reaction, PML = promyelocytic leukemia nuclear body proteins, SLA/LP = antibodies against soluble liver antigen/liver-pancreas, VDRL = venereal disease research laboratory, VZV = varicella-zoster virus.

The patient received 6 cycles of plasmapheresis and IV acyclovir for 53 days. She was extubated and progressively recovered her hepatic function until she did not require any blood-derived transfusion. Her mental status recovered without any sequelae.

There was no history of recurrent infection, and tests that included flow cytometry using markers specific for T, B, and NK cells, immunoglobulin, and complement levels were normal. There was no chronic disease or infection. HIV and tuberculosis tests were negative, and there was no vitamin deficiency. Finally, fulminant hepatitis due to a disseminated HSV-2 primary infection in an immunocompetent host was diagnosed.

Transaminases progressively decreased. However, a cholestatic pattern persisted, wich took up to 6 to 12 months to normalize (data not shown). This was interpreted as cholestatic liver dysfunction. In the periodic follow-up to this day, the patient remains asymptomatic and has normal liver function tests.

This study was approved by Hospital Clínico Universidad de Chile Ethics Committee (N° 58/20). Written informed consent was obtained from the patient.

## 3 Discussion

Identifying the etiology of ALF is essential to guide treatment and establish proper guidelines. In developed countries, acetaminophen toxicity, drug-induced liver injury, ischemia, hepatitis B virus, and autoimmunity account for almost 80% of ALF cases.^[5]^ Prevalence and etiologies are rarely described in the Latino-American adult population.^[4]^

First described in 1969,^[11]^ herpes simplex hepatitis remains a potentially lethal cause of acute necrotizing hepatitis. HSV accounts for 0.8% of ALF cases and 1% to 2% of viral hepatitis cases; however, its mortality rate remains as high as 90% in some reports.^[10]^ It has been mainly documented in immunosuppressed patients, pregnant women, and neonates; however, there are reports of HSV in previously healthy adults.^[9,12]^ In the largest review of HSV hepatitis, 24% of the 137 patients with HSV were considered immunocompetent.^[13]^ Recently, it was described as a complication in patients with severe COVID-19 treated with immunosuppressive therapy.^[14]^

Early treatment with acyclovir can inhibit viral replication and improve outcomes.^[15]^ Unfortunately, the diagnosis of HSV is usually late. In 1 series, the diagnosis of HSV hepatitis was considered antemortem in only 33% of pregnant patients and in 26% of nonpregnant patients^[13]^ Therefore, having high clinical suspicion and identifying some key elements is essential for early diagnosis, as in this case.

### 3.1 Suspicion and diagnostic clues in HSV hepatitis

The diagnosis of herpes hepatitis is challenging and often not straightforward due to the lack of specific signs or symptoms. Identifying some clinical features could raise clinical suspicion and allow physicians to prescribe proven treatments, such as antiviral therapy, early in the disease course.

HSV 1 and 2 belong to the Herpesviridae family; both are common infections worldwide and are mainly known for their presentation as oral or genital ulcers and pharyngitis in adults. The seroprevalence of HSV-2 in the United States is as high as 16% among patients aged 14 to 49 years.^[7]^ VHS presentation is highly variable and depends on whether the infection is primary, nonprimary, or recurrent. Primary infections have a wide range of clinical features, ranging from absent symptoms to severe symptoms with a poor prognosis

The average incubation period after exposure was 4 days (2–12 days).^[16]^ Apart from the classic painful and itchy mucocutaneous lesions, relevant clinical features include systemic features, such as fever, myalgias, malaise, and headaches in 67%, tender lymphadenopathy in 80%, and dysuria in 67% of patients.^[17]^ “Flu-like” symptoms have also been associated with HSV hepatitis.^[13]^

Our patient presented with painful mucocutaneous lesions associated with lymphadenopathy, raising suspicions of HSV hepatitis. This is not a universal finding, and less than half of the reported cases present with mucocutaneous lesions suggestive of HSV.^[10]^

Abdominal pain with clinical or imagenological hepatomegaly is common.^[18]^ Abdominal computed tomography or magnetic resonance imaging findings are nonspecific but may show hepatomegaly, along with edema and diffuse hypodense lesions of 1 to 4 mm, which represent foci of acute hepatic necrosis.^[8]^ Both hepatomegaly and necrotic foci were observed in our case.

General laboratory tests are also nonspecific; leukopenia, lymphopenia, thrombocytopenia, elevated acute phase reactant, coagulopathy, and acute kidney failure are commonly seen in HSV patients.^[18]^ Increased transaminase levels and altered liver function tests, which are hallmarks of ALF, are other clues that establish clinical suspicion. The degree of transaminase elevation can narrow down the list of possible causes during differential diagnoses. Elevations greater than 10 times the upper limit of baseline values are suggestive of ischemic, toxic, or viral liver injury.^[10]^ Moreover, 90% of patients with HSV hepatitis have a 100 to 1000-fold increase in transaminases,^[8]^ particularly with an “anicteric pattern,” indicating the considerable elevation in transaminases, with AST values greater than ALT values and normal or relatively low bilirubin.^[19]^ Our patient presented with more than a 100-fold increase in transaminases, with AST values greater than ALT values and an anicteric pattern. Hepatitis B virus can also present with an anicteric pattern, but ALT values tend to be greater than AST values. Hepatitis A virus could present with markedly elevated ALT and AST values, but it is also usually accompanied by jaundice, with total bilirubin <10 mg/dL.

Primary infection is associated with higher viremia, and serum HSV PCR tests have been shown as more discriminating than serologic tests for diagnosing or excluding HSV as a cause of ALF.^[20]^ The positive HSV PCR test in this case supports the suspicion of primary infection. However, despite these new diagnostic tools, liver biopsy remains the gold standard for diagnosing HSV hepatitis.

Our patient presented with ALF, pharyngitis with painful ulcers, tender lymphadenopathy, and an anicteric liver pattern. Her past history was remarkable for no toxic exposure but unprotected sexual behavior; these factors raised the suspicion of a viral and sexually transmitted disease etiology, particularly HSV.

Interestingly, the development of HSV hepatitis following a surgical procedure has been described.^[21]^ Yokoi et al. reviewed 14 cases with HSV ALF after a surgical procedure; of these cases, 2 were gynecological, where the common presentation was fever, with a median postoperative time of 4.5 days.^[22]^

Drug-induced liver injury (DILI), particularly idiosyncratic hepatotoxic reactions, could resemble symptoms and course of an acute viral hepatitis.^[23]^ This patient consumed amoxicillin–clavulanate (AC) 9 days before the onset of symptoms, therefore AC DILI should be considered in the differential diagnosis. AC- DILI is the most common cause of nonacetaminophen DILI.^[24]^ Median number of days to symptom onset after drug initiation is 29 days (17–37), but shorter latency periods have been described.^[25]^ AC causes DILI more often in men and people over 50 years old. The clinical presentation usually includes jaundice and pruritus. In the laboratory, the most common feature is a cholestatic or mixed pattern of liver function test. Hepatocellular presentation occurs only in 25% of cases,^[24]^ with ALT prominently increased by a factor of 5 to 50.^[23,26]^ Despite its relatively high frequency, severe presentation is rare.^[24]^ When it does occur, severe cases present with marked increase in transaminases and total bilirubin.^[24,27]^ Histology usually shows cholestatic patterns of injury or cholestasic hepatitis. Marked tissue eosinophilia and others immuno-allergic features are common.^[24]^ The characteristics of our patient, the latency of presentation with low levels of bilirubin and markedly elevated transaminases, without the classic findings in the biopsy, led us to conclude that AC DILI was very unlikely.

While it was unlikely that the initial presentation was due to DILI. She evolved with a cholestatic pattern that took months to normalize. Our patient had a prolonged ICU stay with many of the known risk factors for cholestatic liver dysfunction: use of invasive mechanical ventilation, vasopressors, parenteral nutrition, and a wide range of drugs and antibiotics for infectious complications and gram-negative sepsis, in addition to a pre-existing liver damage.^[28]^ However, it cannot be ruled out that some degree of cholestatic DILI secondary to any of the multiple drugs used (particularly antibiotics) was further developed.

### 3.2 Antiviral treatment and the role of plasmapheresis

ALF due to HSV is one of the few diseases with a proven treatment. As discussed previously, the main issue of treating ALF due to HSV is starting antiviral treatment early in the disease course.^[15]^ Patients who received early antiviral treatment displayed a reduction in mortality or the need for liver transplantation (LT).^[13]^

Acyclovir is the most widely used antiviral agent and is a nucleoside analog that is phosphorylated into its active form by thymidine kinase in HSV-infected cells. Acyclovir resistance is rare in immunocompetent patients (<1%), but up to 5% to 25% of immunosuppressed patients resisted acyclovir.^[29]^

The expected mortality according to previous published experience with ALF due to HSV varies between 50% to 90%.^[10,13]^ The differences are partly explained by the percentage of patients promptly treated with acyclovir, transplant patients, and support measures used in the different series. The highest mortality has been reported in non-transplanted patients not treated or treated lately with acyclovir.^[13]^ Several variables, present in our patient, have been statistically associated with a higher risk of death in ALF due to HSV such as: coagulopathy, encephalopathy and platelet count <75,000 U.^[13]^ Although our patient did not meet the classic King's College criteria, she met the Clichy/Villejuif criteria, making her a candidate for liver transplantation. This translates into a probability of spontaneous recovery less than 20%.^[5,30,31]^

Although LT is the standard treatment option once ALF is established, there is growing evidence regarding the benefit of plasmapheresis in such settings.^[32]^ Exchanging plasma may have beneficial effects by removing cytokines and other inflammatory mediators, replacing plasma factors, and modulating the immune system. Different studies have shown positive changes in physiological parameters^[33]^ (cerebral perfusion pressure, hepatic blood flow, coagulopathy, among others); however, the effect of exchanging plasma on relevant clinical outcomes remains unclear. Interestingly, Larsen et al, in a randomized trial that included 182 patients, demonstrated statistically significant LT-free survival benefit with high-volume therapeutic plasma exchange (TPE). The study included 11 patients with ALF and acute viral hepatitis; patients with paracetamol-induced ALF had a better prognosis compared with patients with ALF due to other etiological causes. Moreover, intervention prior to LT did not improve survival compared with patients who received standard medical therapy alone.^[34]^ Actual guidelines recommend TPE in patients with ALF with a recommendation grade of 2B;^[32]^ TPE might be used as a bridge to LT.

Holt et al previously reported 1 successful TPE experience in patients with ALF due to HSV.^[35]^ Potential mechanisms may include the reduction of viral loans and pathogenic viral particles, an effect on humoral immunity, and the removal of nonspecific inflammatory mediators.^[33]^ To our knowledge, this is the second published report of TPE used in patients with HSV ALF.

## 4 Conclusion

ALF is a life-threatening condition, and HSV as an etiology must be suspected based on background, clinical manifestation, and laboratory information. When HSV is suspected, antiviral treatment must be initiated early in the disease course. Further studies are needed to determine the exact role of TPE in patients with ALF due to HSV; however, TPE should be considered especially when LT is limited or contraindicated.

## Acknowledgments

We would like to thanks Jeannette Dabanch for her contribution in the diagnostic process. We declare that we have writing assistance from Editage

## Author contributions

**Conceptualization:** Sebastián M Chávez, Jaime M Poniachik, Rodrigo Cornejo.

**Supervision:** Álvaro M Urzua, Juan P Roblero, Máximo J Cattaneo, Laura E Carreño.

**Writing – original draft:** Sebastián M Chávez, Andrea P Jimenez.

**Writing – review & editing:** Sebastián M Chávez, Jaime M Poniachik, Rodrigo Cornejo.
